# Differentiation of SARS-CoV-2 Variants Using RT-qPCRs by Targeting Recurrent Mutation Sites: A Diagnostic Laboratory Experience from Multi-Center Regional Study, August 2020–December 2021, Poland

**DOI:** 10.3390/ijms23169416

**Published:** 2022-08-20

**Authors:** Karolina Wegrzynska, Magdalena Komiazyk, Jaroslaw Walory, Aleksandra Kozinska, Izabela Wasko, Anna Baraniak

**Affiliations:** Department of Biomedical Research, National Medicines Institute, 00-725 Warsaw, Poland

**Keywords:** COVID-19, SARS-CoV-2, mutations, RT-qPCR variant differentiation, Alpha, Delta, Kappa, Omicron

## Abstract

Rapid identification of SARS-CoV-2 variants is essential for epidemiological surveillance. RT-qPCR-based variant differentiation tests can be used to quickly screen large sets of samples for relevant variants of concern/interest; this study was conducted on specimens collected at 11 centers located in Poland during routine SARS-CoV-2 diagnostics between August 2020 and December 2021. A total of 1096 samples (with CT < 30) were screened for Alpha, Beta, Delta, Kappa and Omicron variants using commercial assays targeting repeat mutation sites. Variants were assigned to 434 (39.6%) specimens; the remaining 662 (60.4%) samples were not classified (no tested mutations detected). Alpha (*n* = 289; 66.59%), Delta (*n* = 115; 26.5%), Kappa (*n* = 30; 6.91%) and Omicron (*n* = 2; 0.46%) variants were identified and their distribution changed over time. The first Alpha variant appeared in October 2020, and it began to gradually increase its proportion of the virus population by June 2021. In July 2021, it was replaced by the Delta variant, which already dominated by the end of the year. The first Kappa was detected in October 2021, while Omicron was found in December 2021. The screening of samples allowed the determination of epidemiological trends over a time interval reflecting the national COVID-19 waves.

## 1. Introduction

The ongoing more than two years pandemic of coronavirus disease 2019 (COVID-19) caused by coronavirus 2 of the severe acute respiratory syndrome (SARS-CoV-2) constitutes a major global health concern [1]. Since the first identification of SARS-CoV-2 in December 2019 [2], the World Health Organization has recorded more than 560 million confirmed cases of COVID-19, including over 6 million deaths [3]; this pandemic is not only a serious public health problem but also an economic and social one [4]. In addition, COVID-19 remains a major epidemiological challenge due to the worldwide spread of SARS-CoV-2 and because the continued evolution of this virus indicates that it is likely to be around for many years to come [5,6].

Epidemiological investigations are increasingly using genome sequence data of disease-causing pathogens. For the first time, such a large amount of genome sequence data on an etiological agent of illness was generated and made publicly available just after the actual emergence of SARS-CoV-2 and its variants [5,6,7]. Under conditions of neutral genetic drift, the rate of SARS-CoV-2 evolution is estimated to be approximately 1 × 10^−3^ mutations/nucleotide yearly [8]. Although most mutations are insignificant, there are also some that confer selective benefits, e.g., related to changes in binding to host cell receptors, and this results in increased infectivity and transmissibility, as well as weak antigen-antibody interaction, reduced neutralization and finally, immune escape [9]; they are so advantageous that, through adaptive evolution within the host, they can increase their frequency in the overall viral population, leading to new lineages. The emergence of SARS-CoV-2 mutants with features dangerous to public health has attracted attention since late 2020; they have been classified as a variant of interest (VOI) and a variant of concern (VOC) in order to establish global surveillance and research efforts. These variants have specific multiple changes to the Wuhan-Hu strain isolated in China during the first outbreak in late 2019. The mutations may make them insensitive to vaccines and can lead to increased infectivity, risk of hospitalization and reduced treatment efficacy, as well as causing diagnostic failures [6,10]. These effects are mainly related to changes occurring in the SARS-CoV-2 spike (S) protein [11].

Among the most relevant variants, the highlighted ones are Alpha (lineage B.1.1.7), Beta (lineage B.1.351), Gamma (lineage P.1), Delta (lineage B.1.627.2) and Omicron (lineage B.1.1.529). All these variants belonged to the VOCs and spread worldwide [6,10,12]. The Alpha variant appeared for the first time in late September 2020 in the United Kingdom (UK) [13], and by the end of the year, it had become the dominant lineage in this country [14]; this variant presents three mutations of interest (N501Y, D614G and P681H) in the S protein [10]. The N501Y alteration increases the affinity of the virus for the host cell ACE2 receptor, and the D614G and the P681H mutations are responsible for enhanced viral transmission [15]. The Beta variant was first identified in October 2020 in South Africa and soon became the dominant strain, constituting the second wave in the region [16]; this variant contains five substitutions of interest in the S protein (K417N, E484K, N501Y, D614G and A701V), three of which are the same as in the Alpha [10]. The K417N and the E484K mutations increase the binding affinity to human ACE2, and the A701V affects antibody neutralization [15,17]. The Gamma variant was initially detected in Brazil [18] and Japan [19]; this variant is characterized by five mutations of interest in the S protein (K417T, E484K, N501Y, D614G H655Y), which, in addition to the last one, are also present in the Beta [10]. The H655Y change enhances the transmissibility of the virus [20]. The Delta variant was first found in India in December 2020 [21], and it includes four substitutions of interest in the S protein, three new ones, L452R and T478K and P681R, and the D614G earlier observed in Alpha, Beta and Gamma [10]. These mutations reduce the ability to neutralize antibodies to recognize the virus and block infection [22]. The Omicron variant was identified for the first time in November 2021 in South Africa [23]. There are several dozen mutations of interest in the S protein in the Omicron sub-lineages. Some of them have been previously identified in the other VOCs [10,23]. The Omicron has been documented in epidemiological studies to be associated with an increased risk of re-infection [24].

The identification of SARS-CoV-2 variants emphasized the need to develop widely available methods to detect and track mutations. Next-generation sequencing (NGS) has been recognized as the gold standard for screening SARS-CoV-2 variants [25]. Although NGS can characterize a variant with the highest accuracy, its implementation is time and labor-consuming, relatively expensive and difficult to access in most medical laboratories. In contrast, commercial PCR-based assays to identify mutations for variant differentiation are rapid, simple to perform, not too costly and therefore, can be used in routine diagnostics [26].

The aim of the study was a retrospective analysis of SARS-CoV-2 positive samples identified at the National Institute of Medicine (NMI) to differentiate virus variants using RT-qPCR kits targeting repeat mutation sites. The study focused on the determination of (i) the time of first appearance of the variant, (ii) the prevalence of variants, and finally, (iii) the changes in SARS-CoV-2 epidemiology over almost 1.5 years.

## 2. Results

### 2.1. Study Specimen Characteristics

#### 2.1.1. Background Analysis

A total of 33,780 clinical samples from hospitals (H), mobile collection sites (MCS) and epidemiological surveillance of individuals in home isolation/quarantine (I/Q) were obtained between 1 August 2020 and 31 December 2021; this corresponded to 17,025 (50.4%) and 16,755 (49.6%) specimens during the study period of the year, respectively. The characteristics of the sample collection are shown in Table 1.

A total of 3217 (9.52%) SARS-CoV-2 positive cases were recognized; this included 2444 (14.36%) and 773 (4.61%) specimens recovered in 2020 and 2021, respectively. The most of them noticed in November 2020 (*n* = 1029; 19.17%), October 2020 (*n* = 758; 14.57%) and March 2021 (*n* = 246; 13.28%). The prevalence of SARS-CoV-2 positive cases from the particular centers varied with *n* = 99 (3.17%) for H1, *n* = 98 (21.97%) for H2, *n* = 698 (12.31%) for H3, *n* = 22 (7.12%) for H14, *n* = 164 (22.34%) for H5, *n* = 8 (9.2%) for H6, *n* = 62 (31.96%) for H7, *n* = 746 (52.57%) for MCS1, *n* = 223 (6.81%) for MCS2, *n* = 303 (17.63%) for I/Q1 and *n* = 21 (44.68%) for I/Q2 during the test period of 2020. Next year, it was *n* = 90 (1.18%), *n* = 54 (4.1%), *n* = 81 (9.57%), *n* = 9 (1.76%), *n* = 86 (10.94%), *n* = 0 (0%), *n* = 91 (20.04%), *n* = 216 (32.1%), *n* = 121 (2.95%), *n* = 8 (1.96%) and *n* = 17 (34%) from the center/collection, respectively.

#### 2.1.2. Final Collection

The final collection (specimens with all tested viral gene cycle thresholds below thirty, CT < 30 as recommended by the test manufacturer) contained 1096 samples in total (34% of all positive cases in the range of CT = 40) (Table 1); it included 589 and 507 SARS-CoV-2 positive cases in 2020 and 2021, which corresponded to 24.1% and 65.59% of all positive specimens during the study periods, respectively. In 2020, this accounted for *n* = 27 (27.27%), *n* = 27 (27.55%), *n* = 147 (21.06%), *n* = 9 (40.91%), *n* = 40 (24.39%), *n* = 3 (37.5%), *n* = 16 (25.81%), *n* = 169 (22.65%), *n* = 30 (13.45%), *n* = 111 (36.63%) and *n* = 10 (47.62%) from the center/collection, respectively. The following year, it was *n* = 65 (72.22%), *n* = 12 (22.22%), *n* = 62 (76.54%), *n* = 8 (88.89%), *n* = 46 (53.49%), *n* = 0 (0%), *n* = 47 (51.65%), *n* = 147 (68.06%), *n* = 104 (85.95%), *n* = 2 (25%) and *n* = 14 (82.35%) in each facility, respectively.

#### 2.1.3. Demographics of the Individuals

The characteristics of the final study cohort are shown in Table 2. The patient group consisted of 554 females (50.55%) and 542 males (49.45%) ranging in age from 0 to 94 years, with a median age of 48 years.

The most common group were individuals aged 36–64 with 502 (45.8%) (*n* = 270 and *n* = 232 in 2020 and 2021, respectively), followed by those aged 19–35 with 256 (23.36%) (*n* = 153 and *n* = 103 in 2020 and 2021, respectively), ≥65 with 254 (23.18%) (*n* = 124 and *n* = 130 in 2020 and 2021, respectively), 6–18 with 52 (4.75%) (*n* = 28 and *n* = 24 in 2020 and 2021, respectively) and 0–5 with 32 (2.92%) cases (*n* = 14 and *n* = 18 in 2020 and 2021, respectively).

### 2.2. Identification of SARS-CoV-2 Variants

A set of 1096 samples were tested for variant differentiation. Results of this analysis are shown in Table 3.

**Figure 1:**
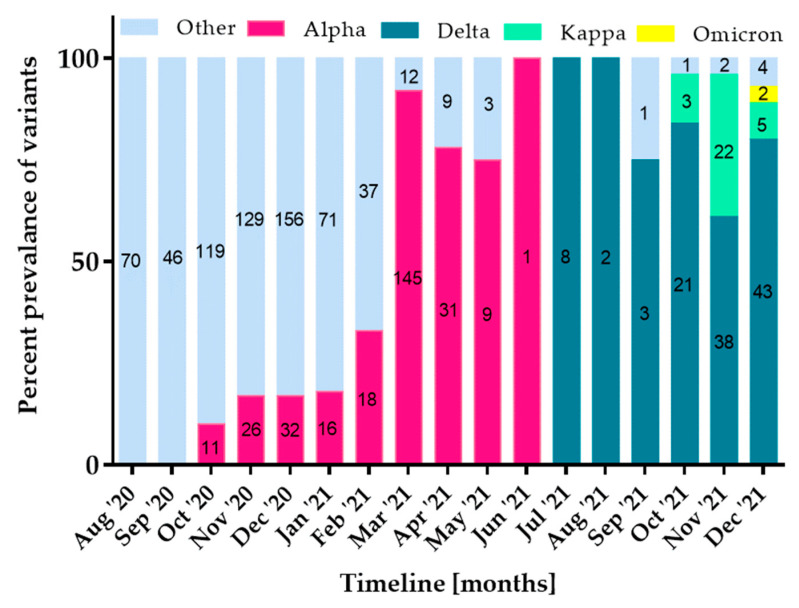
The timeline of distribution of SARS-CoV-2 variants. The light blue color represents specimens for which the tested mutations were not identified, red corresponds to Alpha, dark blue—Delta, green—Kappa, yellow—Omicron variant. The values in the columns indicate the number of samples.

The study was performed for *n* = 70, *n* = 46, *n* = 130, *n* = 155, *n* = 188, *n* = 87, *n* = 55, *n* = 157, *n* = 40, *n* = 11, *n* = 1, *n* = 8, *n* = 2, *n* = 4, *n* = 25, *n* = 62 and *n* = 54 samples from each month over almost 1.5 years. The monthly distribution of SARS-CoV-2 variants is shown in Figure 1 and Table 4.

In total, 436 (39.78%) specimens were assigned a variant. The remaining 660 (60.22%) samples were not classified (no tested mutations detected). Alpha (*n* = 289; 66.59%), Delta (*n* = 115; 26.5%), Kappa (*n* = 30; 6.91%) and Omicron (*n* = 2; 0.46%) variants were detected in the tested specimens; the Beta variant was not identified. There was no statistical significant relationship between gender of patients in whom variants were detected (female, *n* = 554/1096 vs. male, *n* = 542/1096; *p* = 0.767).

Only mutations associated with the Alpha variant were detected in 69 (11.71%) specimens from 2020. The remaining 520 (88.26%) samples were not categorized. The first case of the Alpha variant was identified in a sample collected in 21 October 2020 from a patient hospitalized in H3.

Most cases of the Alpha variant were recorded in MCS1 (*n* = 30; 17.75%), but the highest percentage was in H2 (n = 11; 40.74%). In both hospital and outpatient samples from 2020, the amount of this variant was similar (*p* = 0.569) with *n* = 34/269 (12.64%) and *n* = 35/320 (10.94%) specimens, respectively.

A variety of variants were detected in specimens collected in 2021. Specific mutations were detected for 365 (72%) samples. The remaining 142 (28%) specimens were not classified. The Alpha variant was dominant and represented 220 (43.4%) probes. Between March and June 2021, the variant accounted for 92.36% (*n* = 145), 77.5% (*n* = 31), 75.% (*n* = 9) and 100% (*n* = 1) of the tested specimens in this time interval, respectively. Most cases of the Alpha variant were reported in MCS1 with *n* = 100 and MCS2 with *n* = 35 (68.03% and 33.65% of all samples from these centers, respectively) and the majority of them were collected in March (*n* = 88; 88% and *n* = 24; 72.73%, respectively); however, in H2 hospital the variant was present in 83.33% (*n* = 10) of the studied probes from this center.

The Alpha variant was replaced by the Delta variant in July 2021. A total of 115 (22.7%) samples represented the Delta variant. The first case of this variant was detected in a specimen recovered in 9 July 2021 from patient hospitalized in H3; this variant appeared in samples from several hospitals: H1(*n* = 34; 52.31%), H2 (*n* = 2; 16.67%), H3 (*n* = 22; 35.48%) and H5 (*n* = 9; 19.57%), mobile collection site MCS2 (*n* = 44; 42.31%), and epidemiological surveillance center I/Q2 (*n* = 4; 28.57%). The percentage of Delta variant in relation to other variants present in particular center was the highest in H1 (52.31%), H3 (35.48%) and MCS2 (42.31%). 27.92% (*n* = 67/240) of all hospital cases included in the study from 2021 were identified as Delta variant, while the non-hospital samples were only 17.98% (*n* = 48/267) (*p* = 0.034).

The first case of the Kappa variant was identified in sample taken 7 October 2021 from patient hospitalized in H1. A total of 30 (5.92%) samples represented this variant. The variant occurred at a frequency of 12%, 35.48% and 9.26% from October to December 2021, respectively; it appeared in few hospitals: H1 (*n* = 5; 7.69%), H3 (*n* = 10; 16.13%) and H5 (*n* = 1; 2.17%), and outpatients from MCS2 (*n* = 11; 10.58%) and I/Q2 (*n* = 3; 21.43%). Mutation specific to the Kappa variant accounted for 5.92% of all variant-differentiated samples from 2021.

Only two cases of the Omicron variant were reported in the study, both in December 2021 in MCS2; they consisted 1.92% of the mutation-tested samples in this center and 0.4% of all from 2021. The first one was identified in a specimen collected in 13 December 2021. 

## 3. Discussion

The vaccination is one of the most effective ways to protect from infectious diseases. The development of vaccines against COVID-19 proved successful in preventing symptomatic infection and illness; however, despite the availability of vaccines, many countries experienced multiple COVID-19 waves, largely caused and driven by emerging new VOCs [27]; they prolong the continuation of the disease, leading to patient deaths, economic losses and a further burden on public health systems [4]. Globally, five COVID-19 waves were observed during the study period, from 1 August 2020 to 31 December 2021 [3]. The approximate peak disease periods are July–August 2020 (first wave), October 2020–January 2021 (second wave), March–April 2021 (third wave), July–August 2021 (fourth wave) and finally November–December 2021 (fifth wave with a peak in January 2022). While the first wave was due to the spread of a wild type (WT) of SARS-CoV-2, the following ones were caused by the emergence and dissemination of further different VOCs. 

The situation was slightly different in Poland, where three waves of COVID-19 were observed at the time [28]. The first illness peak appeared in October–November 2020 (first wave), followed by another in February–March 2021 (second wave), and then October–December 2021 (third wave). The number of positive SARS-CoV-2 cases identified during the routine diagnostics in the NMI reflected a nationwide trend, with peaks in the first and second waves, as we already noted in our previously report [29]. A renewed increase in the number of positive samples occurred in the autumn/winter period (full data not shown, partial data in Figure 1), coinciding with the time of the third wave of COVID-19 in the country. A total of 3217 SARS-CoV-2 positive cases were detected, of which 1096 (34%) samples were included in the retrospective study. The final collection was represented by 589 (24.1%) and 507 (65.59%) specimens from 2020 and 2021, respectively. These data indicate that fewer patients in the first year of the study were diagnosed in the active phase of infection with high viral load (CT < 30) than in the second one; this result seems to be related to the health care burden, delays in diagnosis, and the availability of tests for SARS-CoV-2; however, it may also be due to the fact that SARS-CoV-2 was a new virus in the human population in 2020, so people attributed the initial symptoms of infection to other diseases, and therefore got examined later. A statistically significant majority of the tested samples were from adults (*p* < 0.0001) in roughly the same number of each gender, which is consistent with COVID-19 population studies [30,31]. Some papers report that males present an increased risk of severe acute respiratory syndrome infection and higher mortality [32,33]. In our study, we could not confirm this information because the laboratory did not possess clinical data to follow the diseases and their outcomes.

There are only few publications tracing SARS-CoV-2 variants in Poland [29,34,35,36,37]. Most of them based on analysis of viral genomic data deposited at the Global Initiative on Sharing All Influenza Data (GISAID) [5]; it currently contains 83,764 sequences from Poland [5]. Although the NGS is the gold standard for differentiating SARS-CoV-2 variants, due to its long turnaround time and high cost per sample, its use is less feasible by diagnostic laboratories [26]. Therefore, RT-qPCR assays detecting VOCs-specific mutations appear to be a better option for timely clinical applications and population surveillance [26,38,39,40,41]. The present study applied just this method to test a set of more than 1000 samples for SARS-CoV-2 variants. Most of the specimens from 2020 were not classified and likely represented the WT virus. These results are consistent with national data, where the original virus is assigned to the first wave of COVID-19 in the country [42]. The first VOC, Alpha variant, was identified in a sample collected in October 2020. Interestingly, previous data reported that this variant appeared in Poland in December 2020 [37,42]. Since its emergence, the Alpha variant has gradually increased its proportion in the following months, as we already observed in our previous survey [29]. Between March and June 2021, the variant accounted for more than 92% of all recognized cases; this finding also reflects national data, in which the Alpha variant was responsible for the second wave of the epidemic [42]. In July, it was replaced by the Delta variant (the first case identified at the beginning of the month) and was the most commonly identified variant until the end of 2021 (although, it should be noted that the May–September period was represented by a small number of samples). Country data indicate that its initial appearance was in April 2021, but similar to our study, it began to dominate from July and caused the third wave of COVID-19 [42]. The Delta variant was slightly more frequent in hospitalized patients than in outpatients, which was also observed by other authors [43,44]. Two cases of the Omicron variant were reported in December 2021. Based on national data, this variant appeared similar to our research in the last month of 2021 and triggered the fourth wave of disease the following year [42]. Of the investigated VOCs in this study, only the Beta variant was not detected, although other reports indicate few cases of it in the country in 2021 [35,42]; however, one assay identified the Kappa variant, which belongs to the VOIs. The first case of this variant was found in October 2021 and it was represented in the sample collection until the end of the year.

Our study shows that screening by RT-qPCR-based variant differentiation tests allows the identification of epidemiological trends (both temporal and variant dominance) reflecting national COVID-19 waves. Although some of the specimens were not classified by this method, it enabled the testing of a large set of samples. Based on the global data of SARS-CoV-2 spread, the non-categorized specimens from 2020 probably belonged to the WT variant; however, for those from 2021, it seems appropriate to call them “other” considering that they may possess some mutations to the Wuhan-Hu strain that were not identified by the applied assays. Potentially, we should also use the term “likely” for the detected variants (e.g., Alpha-like variant), since only specific mutations were found, and their presence and/or absence of other changes was not verified by genome sequencing. Although, as the NGS-based national data indicated, the variant matches were complete.

A limitation of the study was that not all samples were examined, but only those for which the CT values of the analyzed genes in RT-qPCRs were less than 30. Therefore, in the remaining cases, a specific variants were not defined, potentially underestimating their numbers; this is evidenced by our earlier study, that included all positive samples from the mobile collection site from this study (MCS1) [29]; however, it should be noted that the prevalence of the Alpha variant in the overall virus population remained similar in both investigations.

## 4. Materials and Methods

### 4.1. Specimen Collection

SARS-CoV-2-positive samples collected from patients between 1 August 2020 and 31 December 2021 were analyzed. These included inpatients from seven medical centers (4 third- and 3 s-level hospitals; H1–H7), and outpatients from two drive/walk-thru mobile collection sites (MCS1—drive-thru mobile collection site, MCS2—walk-thru mobile collection site) and epidemiological surveillance of individuals in home isolation/quarantine (I/Q1—samples from towns in Mazovia, I/Q2—samples from Warsaw) in Mazovia, Poland. The specimens were nasopharyngeal or combined pharyngeal and nasal swabs obtained by the NMI as part of routine examinations for SARS-CoV-2 infection. The samples were transported in virus-dedicated media, along with the patient’s basic demographics and consent to use the material for scientific research obtained through a laboratory form. All specimens were coded to prevent identification of personal information during the testing, and after the diagnostic process they were stored at −80 °C, pending a retrospective study.

### 4.2. Nucleic Acids Extraction Methods and SARS-CoV-2 Detection 

Virus RNA acids were isolated using two procedures, column-based extraction by Viral DNA/RNA kit (A&A Biotechnology, Gdansk, Poland) and magnetic-based method via NucleoMag Pathogen kit (Machery-Nagel, Duren, Germany) with modifications to the manufacturers’ instructions, as described previously [45].

The MutaPLEX^®^ Coronavirus (SARS-CoV-2) kit (Immundiagnostik AG, Bensheim, Germany) detecting S/RdRP and E genes of SARS-CoV-2 was used for in vitro diagnostics. RT-qPCRs were carried out with Applied Biosystems QuantStudio 6 Pro Real-Time PCR system (Life Technologies Holdings Pte Ltd., Singapore). Both the test parameters and reading rules (based on CT values) were performed according to the test manufacturer’s recommendations.

### 4.3. Differentiation of SARS-CoV-2 Variants 

The samples with CT < 30 in all SARS-CoV-2 genes detected during routine diagnostics were tested for some VOCs identification using three commercial variant-differentiation assays. The tests were carried out according to the manufacturers’ guidelines, using an Applied Biosystems QuantStudio 6 Pro Real-Time PCR System instrument. To determine the time of first emergence of the variant in the study collection, all samples found at least one month before the initial worldwide appearance of the VOC were examined.

#### 4.3.1. Alpha Variant Identification

Samples previously identified as SARS-CoV-2 positive isolated from patients between August 2020 and December 2021 were tested for Alpha using the Bosphore^®^ SARS-CoV-2 Variant Detection Kit v1 assay (Anatolia Geneworks, Istanbul, Turkey); this assay uses appropriately designed primers and detects the A570D, P681H and Y144del mutations that have been observed in the UK variants.

#### 4.3.2. Beta, Delta and Kappa Variants Identification

The specimens recovered from patients between September 2020 and December 2021 were tested for detection of the Beta, Delta and Kappa variants. The ID™ SARS-CoV-2/VOC evolution Pentaplex kit (ID Solutions, Grabels, France) was used to find these variants. The primers used in the assay serve to amplify specific target sequences. In addition to the variant-specific L452R, E484K and E484Q mutations, the kit also identifies unmodified N2, RdRp1 and RdRp2 SARS-CoV-2 genes, which allowed verification of the used samples. The manufacturer’s data show that the limit of detection is 10 copies per PCR reaction for SARS-CoV-2 and K417N mutation and 40 copies/PCR for L452R and E484K mutations.

#### 4.3.3. Omicron Variant Identification

The samples collected from October 2021 to December 2021 were checked for Omicron variant. Detection of this variant was performed using PKamp VariantDetect SARS-CoV-2 RT-PCR KIT Combination 6 (PerkinElmer, Waltham, MA, USA); this kit detects L452R, P681H and P681R mutations using primers and TaqMan probes, which allows the identification of the Alpha and Delta variants in addition to Omicron. Furthermore, it also provides verification of SARS-CoV-2 positive samples by detecting the N/ORF1ab virus genes. The detection limit of this test amounts to 200 copies per PCR reaction for tested mutation.

### 4.4. Statistical Analysis

The statistical analysis of data and figure was performed with GraphPad Prism 7.0 software (GraphPad Software, Inc., San Diego, CA, USA). Two-sided chi-square or Fisher’s exact test was used in *p*-value calculation at alpha < 0.05.

## 5. Conclusions

The present study showed that differentiation of SARS-CoV-2 variants by RT-qPCR is highly effective in retrospective studies of a large set of samples and reflects national epidemiological trends (prevalence and types of virus variants). The obtained results entitle this method to application in real-time VOCs monitoring. Changes in the functional properties of new SARS-CoV-2 variants can lead to differences in the clinical course of the infection. Therefore, rapid identification of such variants in population allows timely introduction of appropriately adjusted prevention and adaptation of medical intervention in the form of an optimal procedure and treatment method.

## Figures and Tables

**Table 1:** Centers included in the study with the numbers of total samples and negative/positive cases recovered at each site in 2020 and 2021.

Center ^1^	Type of Center/ Collection	2020				2021			
		No. of Samples	No. of Negative Cases	No. of Positive Cases	No. of Positive Cases with CT < 30	No. of Samples	No. of Negative Cases	No. of Positive Cases	No. of Positive Cases with CT < 30
H1	third level hospital	3125	3026	99	27	7603	7429	90	65
H2		446	348	98	27	1317	1224	54	12
H3		5671	4973	698	147	846	747	81	62
H4		309	287	22	9	510	498	9	8
H5	second level hospital	734	570	164	40	786	658	86	46
H6		87	79	8	3	0	0	0	0
H7		194	132	62	16	454	344	91	47
MCS1	drive-thru walk-thru	1419	673	746	169	673	435	216	147
MCS2		3274	3051	223	30	4107	3971	121	104
I/Q1	Epidemio-logical Survei-llance	1719	1416	303	111	409	394	8	2
I/Q2		47	26	21	10	50	29	17	14

^1^ H—hospital, MCS—mobile collection site, I/Q—home isolation/quarantine.

**Table 2:** Characteristics of the patients with samples subjected to SARS-CoV-2 variant differentiation by center, gender and age group.

		No. of Tested Samples ^2^										
	Center ^1^	H1	H2	H3	H4	H5	H6	H7	MCS1	MCS2	I/Q1	I/Q2
Variables												
Female		13/36	13/2	77/31	9/7	22/26	3/0	4/23	80/71	14/60	55/2	2/4
Male		14/29	14/10	70/31	0/1	18/20	0/0	12/24	89/76	16/44	56/0	8/10
Age in years:												
0–5		1/10	0/0	5/3	0/2	0/0	0/0	1/1	3/1	0/1	4/0	0/0
6–18		1/10	1/0	8/1	0/0	2/0	0/0	0/0	7/2	0/11	9/0	0/0
19–35		4/15	1/0	27/15	4/4	11/3	1/0	0/1	57/27	10/36	37/0	1/2
36–64		18/28	20/3	56/24	5/2	18/22	2/0	5/19	80/79	19/48	42/2	5/5
≥65		3/2	5/9	51/19	0/0	9/21	0/0	10/26	22/38	1/8	19/0	4/7

^1^ H—hospital, MCS—mobile collection site, I/Q—home isolation/quarantine. ^2^ the numbers of tested samples are separated for 2020 and 2021.

**Table 3:** SARS-CoV-2 variants in each center with the numbers of their samples in 2020 and 2021.

Center ^1^	2020			2021					
	No. of Tested Samlpes	SARS-CoV-2 Variant		No. of Tested Samples	SARS-CoV-2 Variant				
		Alpha	Other		Alpha	Delta	Kappa	Omicron	Other
H1	27	3	24	65	18	34	5	0	8
H2	27	11	16	12	10	2	0	0	0
H3	147	11	136	62	22	22	10	0	8
H4	9	0	9	8	6	0	0	0	2
H5	40	6	34	46	13	9	1	0	23
H6	3	0	3	0	0	0	0	0	0
H7	16	3	13	47	11	0	0	0	36
MCS1	169	30	139	147	100	0	0	0	47
MCS2	30	1	29	104	35	44	11	2	12
I/Q1	111	4	107	2	0	0	0	0	2
I/Q2	10	0	10	14	5	4	3	0	2

^1^ H—hospital, MCS—mobile collection site, I/Q—home isolation/quarantine.

**Table 4:** The monthly prevalence of SARS-CoV-2 variants in particular centers.

Month of Collection	Variant	No. of Variant Samples in Centers ^1^										
		H1	H2	H3	H4	H5	H6	H7	MCS1	MCS2	I/Q1	I/Q2
20 August	Other	0	0	15	0	0	0	0	0	1	54	0
20 September	Other	0	0	17	0	0	0	0	0	5	24	0
20 October	Alpha	1	0	5	0	1	0	0	0	0	4	0
	Other	9	2	38	0	10	3	0	18	8	29	2
20 November	Alpha	1	1	3	0	2	0	0	18	1	0	0
	Other	9	11	37	5	13	0	6	44	2	0	2
20 December	Alpha	1	10	3	0	3	0	3	12	0	0	0
	Other	6	3	29	4	11	0	7	77	13	0	6
21 January	Alpha	3	3	2	1	0	0	2	5	0	0	0
	Other	1	0	5	0	7	0	14	34	7	2	1
21 February	Alpha	0	0	6	1	0	0	2	7	2	0	0
	Other	0	0	2	1	1	0	21	11	1	0	0
21 March	Alpha	8	6	9	1	5	0	2	88	24	0	2
	Other	1	0	0	0	7	0	1	2	1	0	0
21 April	Alpha	4	0	3	3	7	0	3	0	8	0	3
	Other	1	0	1	0	7	0	0	0	0	0	0
21 May	Alpha	3	1	2	0	1	0	1	0	1	0	0
	Other	2	0	0	0	1	0	0	0	0	0	0
21 June	Alpha	0	0	0	0	0	0	1	0	0	0	0
21 July	Delta	1	0	2	0	0	0	0	0	5	0	0
21 August	Delta	1	0	1	0	0	0	0	0	0	0	0
21 September	Delta	1	0	1	0	1	0	0	0	0	0	0
	Other	0	0	0	1	0	0	0	0	0	0	0
21 October	Delta	9	0	0	0	1	0	0	0	11	0	0
	Kappa	1	0	0	0	0	0	0	0	2	0	0
	Other	1	0	0	0	0	0	0	0	0	0	0
21 November	Delta	9	1	12	0	2	0	0	0	11	0	3
	Kappa	2	0	9	0	0	0	0	0	8	0	3
	Other	0	0	0	0	0	0	0	0	2	0	0
21 December	Delta	13	1	6	0	5	0	0	0	17	0	1
	Kappa	2	0	1	0	1	0	0	0	1	0	0
	Omicron	0	0	0	0	0	0	0	0	2	0	0
	Other	2	0	0	0	0	0	0	0	1	0	1

^1^ H—hospital, MCS—mobile collection site, I/Q—home isolation/quarantine.

## Data Availability

All relevant data are within the manuscript.
